# Engineering *Saccharomyces cerevisiae* for the production of dihydroquercetin from naringenin

**DOI:** 10.1186/s12934-022-01937-8

**Published:** 2022-10-15

**Authors:** Shiqin Yu, Mingjia Li, Song Gao, Jingwen Zhou

**Affiliations:** 1 Science Center for Future Foods, Jiangnan University, 1800 Lihu Road, Wuxi, 214122 Jiangsu China; 2 Key Laboratory of Industrial Biotechnology, Ministry of Education and School of Biotechnology, Jiangnan University, 1800 Lihu Road, Wuxi, 214122 Jiangsu China; 3 Engineering Research Center of Ministry of Education On Food Synthetic Biotechnology, Jiangnan University, 1800 Lihu Road, Wuxi, 214122 Jiangsu China; 4 Jiangsu Province Engineering Research Center of Food Synthetic Biotechnology, Jiangnan University, 1800 Lihu Road, Wuxi, 214122 Jiangsu China

**Keywords:** Dihydroquercetin, Bioproduction, *Saccharomyces cerevisiae*, Naringenin

## Abstract

**Background:**

Dihydroquercetin (DHQ), a powerful bioflavonoid, has a number of health-promoting qualities and shows potential as a treatment for a number of disorders. Dihydroquercetin biosynthesis is a promising solution to meet the rising demand for dihydroquercetin. However, due to the significant accumulation of eriodietyol (ERI), naringenin (NAR), dihydrokaempferol (DHK), and other metabolites, the yield of DHQ biosynthesis is low. As a result, this is the hindrance to the biosynthesis of DHQ.

**Results:**

In this study, we proposed several strategies to enhance the product formation and reduce the metabolites in accumulation. The flavonoid 3′-hydroxylase (F3′H) and cytochrome P450 reductase from different species were co-expressed in *S. cerevisiae*, and the best strain expressing the P450-reductase enzyme complex (*Sm*F3′H/*Sc*CPR) yielded 435.7 ± 7.6 mg/L of ERI from NAR in the deepwell microplate. The product conversion rate was improved further by mutating the predicted potential ubiquitination sites to improve SmF3′H stability, resulting in a 12.8% increase in titre using the mutant SmF3′H (K290R). Besides, different F3Hs from various sources and promoters were tested for the improved DHQ production, with the best strain producing 381.2 ± 10.7 mg/L of DHQ from 1 g/L of NAR, suggesting the temporal regulation the expression of F3H is important for maximization the function of F3′H and F3H.

**Conclusion:**

This study offers effective strategies for improving DHQ production from NAR and could be used as a reference for related research.

**Supplementary Information:**

The online version contains supplementary material available at 10.1186/s12934-022-01937-8.

## Introduction

Dihydroquercetin, commonly known as taxifolin, is a bioactive flavonoid found in plants such as milk thistle, red onion, and acai palm, as well as in various commercial preparations like Legalon, Pycnogenol, and Venoruton [1, 2]. DHQ has two stereocenters and four types of stereoisomers: ( +)-taxifolin, (−)-taxifolin, ( +)-epitaxifolin, and (−)-epitaxifolin; and these stereoisomers have different activities and distribution in the body [3]. The biological activities of dihydroquercetin, such as antioxidation, antiviral, and anticancer, render it an important dietary supplement and functional food, offering therapeutic promise in some disease prevention and treatment, such as neurodegenerative diseases, hypertension, and viral infection [2, 3]. Studies have shown that the antioxidant capacity of dihydroquercetin surpasses that of common antioxidants such as tocopherols and ascorbic acid, which is a powerful free radical neutraliser that protects body tissues from harmful free radicals [4]. In both in vivo and in vitro tests, dihydroquercetin has been shown to be effective as a cancer treatment via multiple mechanisms with little or no side effects on normal healthy cells [5]. In recent years, dihydroquercetin has been shown as a promising inhibitor for coronaviruses by inhibiting 3C-like protease [6], and it may be used as an efficient free radical scavenger in the treatment of COVID-19 in the near future [7]. As the need for an effective medicine with few or no side effects grows, this substance has come to light as a possible way to produce better medicine to meet the growing demand.

Currently, DHQ is commonly extracted from the wood of Dahurian Larch, but this tree grows slowly as it thrives in the world's most northern region, where climatic circumstances are exceptionally harsh [8]. Extraction from plant materials is a typically tedious process involving the use of toxic organic solvents [8–10]. Chemical synthesis of ( ±)-taxifolin has been documented, and requires chiral reverse-phase HPLC for chiral separation [11]. Although around 25% of marketed drugs are racemic mixtures, the single-isomer has significant therapeutic importance since one of the isomers could provide more effective treatment, high bioavailability, lower exposure in poor metabolizers, and less interindividual variation [12, 13]. As compared to chemical synthesis, bioproduction is more suited to producing such structurally complicated natural chemicals that involve stereoselectivity, and customers prefer products labelled with "bio". DHQ can be sterespecifically hydroxylating at the C-ring of ERI at the 3′-position or the B-ring of DHK at the 3-position by the enzyme flavanone 3-hydroxylase (F3H), and both of these molecules can be synthesized from NAR, an inexpensive starting material that could be extracted from the fruit-juice industry’s wastes [14]. *Yarrowia lipolytica* was engineered to produce 110.5 mg/L of DHQ from glucose while also accumulating a significant amount of metabolites, including 252.4 mg/L of NAR and 134.2 mg/L of ERI [15]. Recently, the *Escherichia coli* was engineered to convert 100 mg/L of NAR to 13.6 mg/L of DHQ, and the bacterium was further engineered to produce 20.1 mg/L of DHQ from glycerol with a significant accumulation of DHK and NAR [16]. These studies have shown that DHQ can be bioproduced via the key metabolite NAR by further hydroxylation at specific positions, but future research should focus on addressing the problem of significant intermediator accumulations and the low yield of target compound.

Previous research has shown that flavonoid 3′-hydroxylase (F3′H) and flavanone 3-dioxygenase (or flavanone 3-hydroxylase, F3H) are rate-limited steps in NAR hydroxylation [15–17], with F3′H requiring cytochrome P450 reductase for electron transfer. Many F3′Hs and F3Hs have been mined and functionally analyzed [18–21], but no systematic comparison of the activities involved in DHQ production has been conducted. Previous studies have also shown that the optimization of the expression of enzyme and its redox partner could lead to a substantial improvement in product formation. Lv et al. identified that the chalcone synthase (CHS) and cytochrome P450 reductase (CPR) were the bottleneck for the hydroxylated flavonoid production, and optimizing the expression via adjusting the gene copy number of CHS and CPR could improve the fluovonid production [15]. In another case study, CYP450 BM3 varaint was used for the biosynthesis of ERI from NAR, and the best mutant could produce 47 μM from 100 μM in a 3-L bioreactor [22]. Gao et al. significantly increased ERI production from NAR by adjusting promoter strength and mutating the vital enzyme flavonoid 3′-hydroxylase and its redox partner in shaking flasks, producing approximately 1 g/L of ERI from 1.5 g/L of NAR [23]. These results show that increasing the activity of enzymes or improving the expression of F3′H and F3H could lead to a boost in flavovoid synthesis.

In this study, the interaction of F3′H and redox partner cytochrome P450 reductases was optimized to improve substrate conversion, and F3′H stability in cells was improved by modifying the predicted potential ubiquitination sites. Besides, different F3Hs from different sources and different types of promoters were tested for a better performance in DHQ production in the shake flask, and the best strain could produce 381.2 ± 10.7 mg/L of DHQ from 1 g/L of NAR.

## Material and methods

### Strains, plasmids, and primers

All primers were synthesised by GENEWIZ (China) and Sangon Biotech (China), and are listed in Additional file 1: Table S2. The strain of *E. coli* JM109 was used for cloning purposes, and the *S. cerevisia* C800 was derived from *S. cerevisia* CEN.PK2-1D (*MAT*α, *ura*3-52, *trp*1-286, *leu*2-3, 112, *his*3Δ1) by inactivating the gene *gal80*, which was used for gene expression and whole-cell biocatalysis in the study. The plasmid pY26 was used for episomal expression.

### Growth media and culture conditions

*E. coli* strains were cultivated in Erlenmeyer flasks with Luria–Bertani broth in a shaking incubator at 220 rpm and 37 °C. For plasmid construction purposes, medium should be added with a final concentration of 100 μg/mL amplicilin to maintain plasmid stability in liquid broth or solid medium. The constructed plasmids were verified by sequencing (performed by GENEWIZ (China)) and then transformed into the yeast cells following the protocol from the literature [24]. Regarding the selection of recombinant yeast strains, yeast nitrogen base (YNB) was used to prepare the synthetic medium for isolating the auxotrophic mutants, and yeast extract peptone dextrose (YPD) was used for the cultivation of yeast cells. Depending on the purposes, yeast were grown at 30 °C in liquid or solid medium of YNB or YPD.

### Expression plasmid construction

Gene fragments were codon-optimized and synthesised by GENEWIZ (China), and assembled with the plasmid backbone from pY26-TEF-GPD [25] and pRS424 [26] following Gibson assembly. The constructed plasmids were sequenced for verification, and transformed into *S. cerevisiae* for biotransformation. The synthesised genes and constructed plasmids are listed in Additional file 1: Table S1 and Table 1.

**Table 1:** The constructed plamids for optimizing the interaction between F3′Hs and CPRs

Constructed plasmids	Characteristics	Reference
pY26-*SmF3′H*-*SmCPR*	Episomal expression vector, containing the genes of *SmF3′H* and *SmCPR* from *S. marianum*	This study
pY26-*SmF3′H*-*AtCPR*	Episomal expression vector, containing the genes of *SmF3′H* from *S. marianum* and *AtCPR* from *A. thaliana*	This study
pY26-*SmF3′H*-*EbCPR*	Episomal expression vector, containing the genes of *SmF3′H* from *S. marianum* and *EbCPR* from *E. breviscapus*	This study
pY26-*SmF3′H*-*GmCPR*	Episomal expression vector, containing the genes of *SmF3′H* from *S. marianum* and *GmCPR* from *G. max*	This study
pY26-*SmF3′H*-*HtCPR*	Episomal expression vector, containing the genes of *SmF3′H* from *S. marianum* and *HtCPR* from *H. tuberosus*	This study
pY26-*SmF3′H*- *HtCPR*(L1M)	Episomal expression vector, containing the genes of *SmF3′H* from *S. marianum* and *HtCPR* from *H. tuberosus*(replaced the start codon UUG with ATG)	This study
pY26-*SmF3′H*- *HtCPR*(+ M)	Episomal expression vector, containing the genes of *SmF3′H* from *S. marianum* and *HtCPR* from *H. tuberosus*(added with a start codon of ATG)	This study
pY26-*SmF3′H*-*ScCPR*	Episomal expression vector, containing the genes of *SmF3′H* from *S. marianum* and *HtCPR* from *S. cerevisiae*	This study
pY26-*FaF3′H*-*SmCPR*	Episomal expression vector, containing the genes of *FaF3′H* from *F. x ananassa* and *SmCPR* from *S. marianum*	This study
pY26-*FaF3′H*-*AtCPR*	Episomal expression vector, containing the genes of *FaF3′H* from *F. x ananassa* and *AtCPR* from *A. thaliana*	This study
pY26-*FaF3′H*-*EbCPR*	Episomal expression vector, containing the genes of *FaF3′H* from *F. x ananassa* and *EbCPR* from *E. breviscapus*	This study
pY26-*FaF3′H*-*GmCPR*	Episomal expression vector, containing the genes of *FaF3′H* from *F. x ananassa* and *GmCPR* from *G. max*	This study
pY26-*FaF3′H*-*HtCPR*	Episomal expression vector, containing the genes of *FaF3′H* from *F. x ananassa* and *HtCPR* from *H. tuberosus*	This study
pY26-*FaF3′H*-*ScCPR*	Episomal expression vector, containing the genes of *FaF3′H* from *F. x ananassa* and *ScCPR* from *S. cerevisiae*	This study

### Yeast genome edition

The yeast CRISPR/Cas9 system was used to integrate the *Sc*CPR expression cassette into the genome HO site according to the protocol [27]. Briefly, sgRNA was designed based on the analysis from the CRISPR gRNA Design Tool Benchling (https://www.benchling.com/crispr/), and the recombination arms were fused with the *Sc*CPR expression cassette, then transformed into yeast cells with CRISPR/Cas9 plasmids. Mutants were isolated from YNB-based synthetic medium without uracil for selecting yeast mutants. The mutants were counter selected on the medium containing 5-fluoroorotic acid against the URA3 gene to remove CRISPR/Cas9 plasmids.

### Biotransformation in 24 deep-well microplates

The recombinant strains were grown on YNB-URA medium, and the colonies were picked up and inoculated into 10 mL of YNB-URA liquid medium for growing at 30 °C, 220 rpm until they reached the logarithmic phase. The 250 μL of starter was transferred to a fresh YPD medium with the addition of 1 g/L NAR as the substrate. The samples were collected after the strains had grown for 72 h at the conditions of 30 °C, 220 rpm. Similarly, the starter was prepared as mentioned before, and 30 μL of starter was transferred into 3 mL of fresh YPD medium containing 1 g/L of NAR in the 24 deep-well microplate, and samples were taken for analysis after the strain had grown for 72 h at 30 °C, 220 rpm.

### Analytical methods

The optical density was determined by using a microplate reader (Tecan infinite 200 pro, Switzerland), and the samples were diluted in an appropriate range for measurement. The samples of NAR, DHQ, ERI, and DHK were mixed in an equal volume of pure methanol, and the concentrations were analysed using the Shimadzu high-performance liquid chromatography (HPLC) system (Shimadzu Corporation, Kyoto, Japan). The detection was performed using a Thermo Scientific Hypersil ODS-2 C18 column (Thermo Fisher Scientific Inc., USA), and compounds were eluted using a gradient elution protocol that consisted of acetonitrile containing 0.1% trifluoroacetic acid and water at a flow rate of 1.0 mL/min. All the compounds were detected using an ultraviolet detector A370 at 290 nm.

## Results

### Optimization of the expression for flavonoid 3′-hydroxylase and its redox partner

**Fig. 1:**
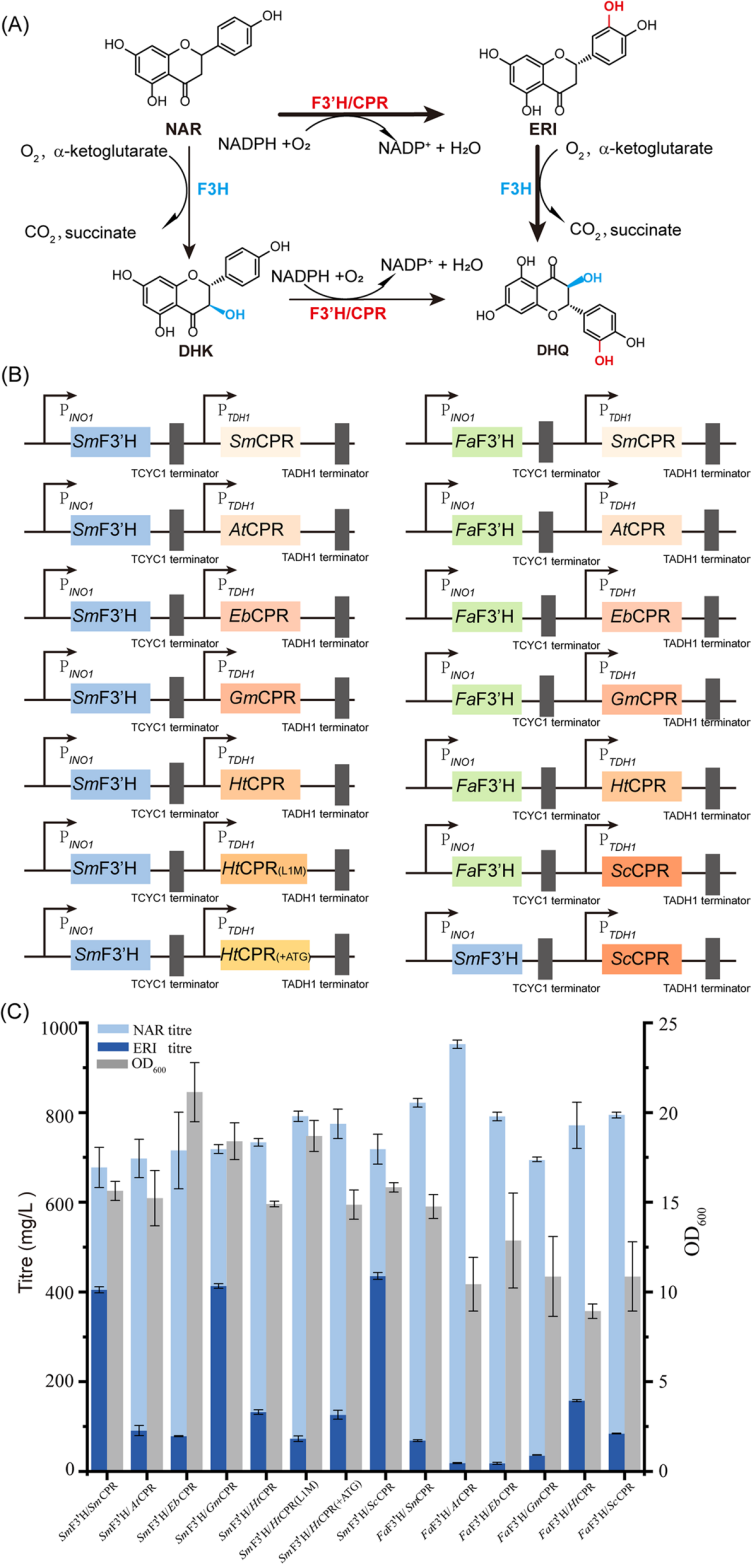
Plasmid construction and optimization of interaction between the flavonoid 3′-hydroxylase and the redox partner. **A** Schematic representation of DHQ bioproduction from NAR. **B** Plasmid construction for F3′H and CPR co-expression. **C** The tests for the optimal enzyme complex for NAR biotransformation. All the genes encoding the F3′Hs and CPR from different sources were controlled under the promoters of P_*INO1*_ and P_*TDH1*_, respectively. For testing the expressed enzyme complex activity, one gram of NAR was used as the substrate and added to the medium YPD. All experiments were performed in triplicates and error bars indicate SD

The hydroxylation of the C-ring and B-ring of flavonoids at the 3- and 3′positions, catalyzed by flavanone 3-dioxygenase (F3H) and flavonoid 3′-hydroxylase (F3′H), is required for the production of DHQ from NAR (Fig. 1A). The F3′Hs are cytochrome P450-dependent enzymes that could hydroxylate the NAR to produce ERI, and this process requires redox partners for electron delivery. Previous studies indicated that the optimization of redox partner interactions with P450 enzymes has shown faster substrate conversion and an increased product yield [28, 29], which may be achieved by optimizing of the expression F3′H and its redox partner to form a more active enzyme complex. In our study, *Silybum marianum* (*Sm*CPR), *Arabidopsis thaliana* (*At*CPR), *Erigeron breviscapus* (*Eb*CPR), *Glycine max* (*Gm*CPR)*, Helianthus tuberosus* (*Ht*CPR)*,* and *S. cerevisiae* (*Sc*CPR), and were episomally co-expressed with F3′Hs from *Sm*F3′H or *Fragaria x ananassain* (*Fa*F3′H) in *S. cerevisiae* (Fig. 1B and Table 1)*.* The constructed recombinant strains were cultivated for enzyme expression in the YPD medium containing 1 g/L NAR as the substrate, and the supernatants were taken after fermentation ran for 72 h. The strains that co-expressed the *Sm*F3′H and CPRs of *Sc*CPR, *Gm*CPR, or *Sm*CPR produced much higher titres of ERI, with the best P450-reductase enzyme complex *Sm*F3′H/*Sc*CPR achieving a titre of 435.7 ± 7.6 mg/L ERI and a yield of 64.1 ± 2.2 mg/g_CDW_, the highest titre and yield in all the experimental groups (Fig. 1C).

The truncation was applied to SmF3′H and ScCPR to investigate the influence of the transmembrane regions. The transmembrane regions of these two proteins were analyzed with the software TMHMM-2.0 and Alphafold for structure prediction (Additional file 1: Fig. S1A), and subsequently deleted to produce the distinct truncated proteins *Sm*F3′H-M1, *Sm*F3′H-M2, and *Sc*CPR-M. The biotransformation of NAR to ERI was not significantly impacted by the deletion of the *Sc*CPR's transmembrane region, while the truncation of *Sm*F3′H nearly eliminated the catalytic activity (Additional file 1: Fig.S1B).

### Improvement of the enzyme stability by mutating the potential ubiquitination sites

**Fig. 2:**
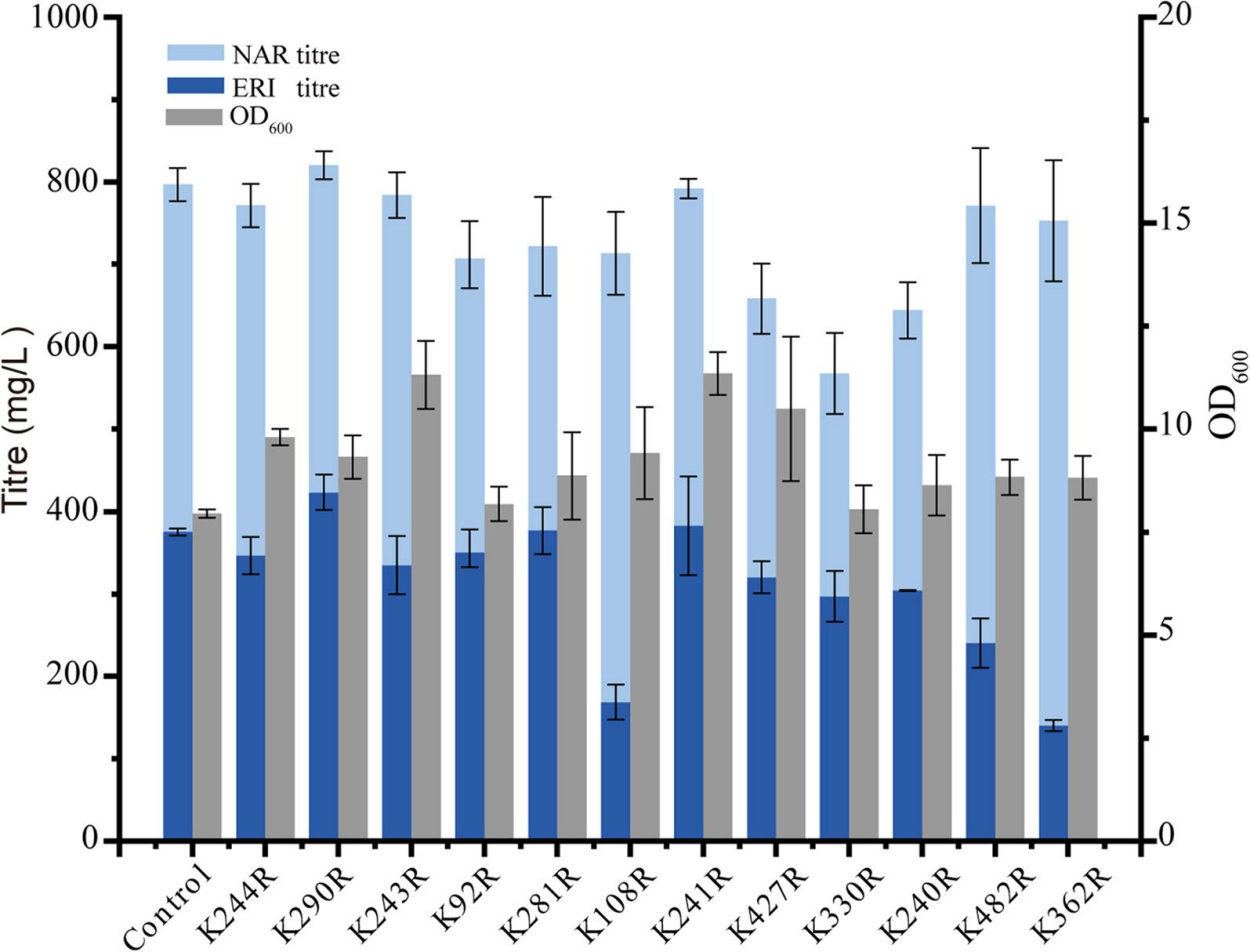
Increasing *Sm*F3′H’s stability by mutating potential ubiquitination sites. The potential ubiquitination sites on the enzyme *Sm*F3′H were mutated based on the predicted results from BDM-PUB, and were episomlly expressed in a *S. cerevisiae* strain that integrated a copy of *Sc*CPR into the genome HO site. The recombination strains were tested for their ability to convert NAR to ERI in a 24 deep-well microplate after 72-h fermentation. All experiments were performed in triplicates and error bars indicate SD

Ubiquitin-mediated proteasomal degradation is one of the main proteolytic pathways for regulating protein stability and turnover depending on cellular needs in eukaryotes, mainly involving the degradation of misfolded and damaged proteins along with the short-lived proteins [30, 31]. A previous study has shown that the mutation at the sensitive ubiquitination site of *Fj*TAL can lead to a substantial improvement in *p*-coumaric acid production [32]. To examine the ubiquitination sites and prolong the protein half life, an online web server for protein ubiquitination sites prediction, BDM-PUB with Bayesian Discrimnant method, was used to identify the potential candidates (Table 2). These potential ubiquitination sites were mutated to arginine residues to create *Sm*F3′H mutants and were episomal expressed in a *S. cerevisiae* strain that integrated a copy of *Sc*CPR into the genome HO site. The recombinant strains were examined for the contribution of *Sm*F3′H mutants in eriodietyol production from naringenin, and the mutant K290R was able to slightly improve *Sm*F3′H performance in the conversion of NAR to ERI, obtaining a 12.8% increase in titre compared with wild type *Sm*F3′H (Fig. 2). Mutations at other predicted ubiquitination sites did not improve the ERI production, and some even resulted in decreased product formation, indicating that neutral or harmful mutations occurred in the enzymes.

**Table 2:** The predicted ubiquitination sites on *Sm*F3′H

Protein	position	Sequence	scorces
*Sm*F3′H	244	KMK**K**LHL	2.13
	290	EGG**K**LSD	1.58
	243	KKM**K**KLH	2.06
	92	QFL**K**VHD	1.90
	281	ISL**K**DDA	1.82
	108	SGA**K**HIA	1.23
	241	VTK**K**MKK	1.08
	427	GGE**K**PNA	1.05
	330	QLL**K**QAQ	3.04
	240	SVT**K**KMK	2.40
	482	DPE**K**LNM	0.51
	362	AIV**K**ETF	2.15

The predicted ubiquitination sites were highlighted in bold

### Selection of optimal flavanone 3-dioxygenase for dihydroquercetin production

**Fig. 3:**
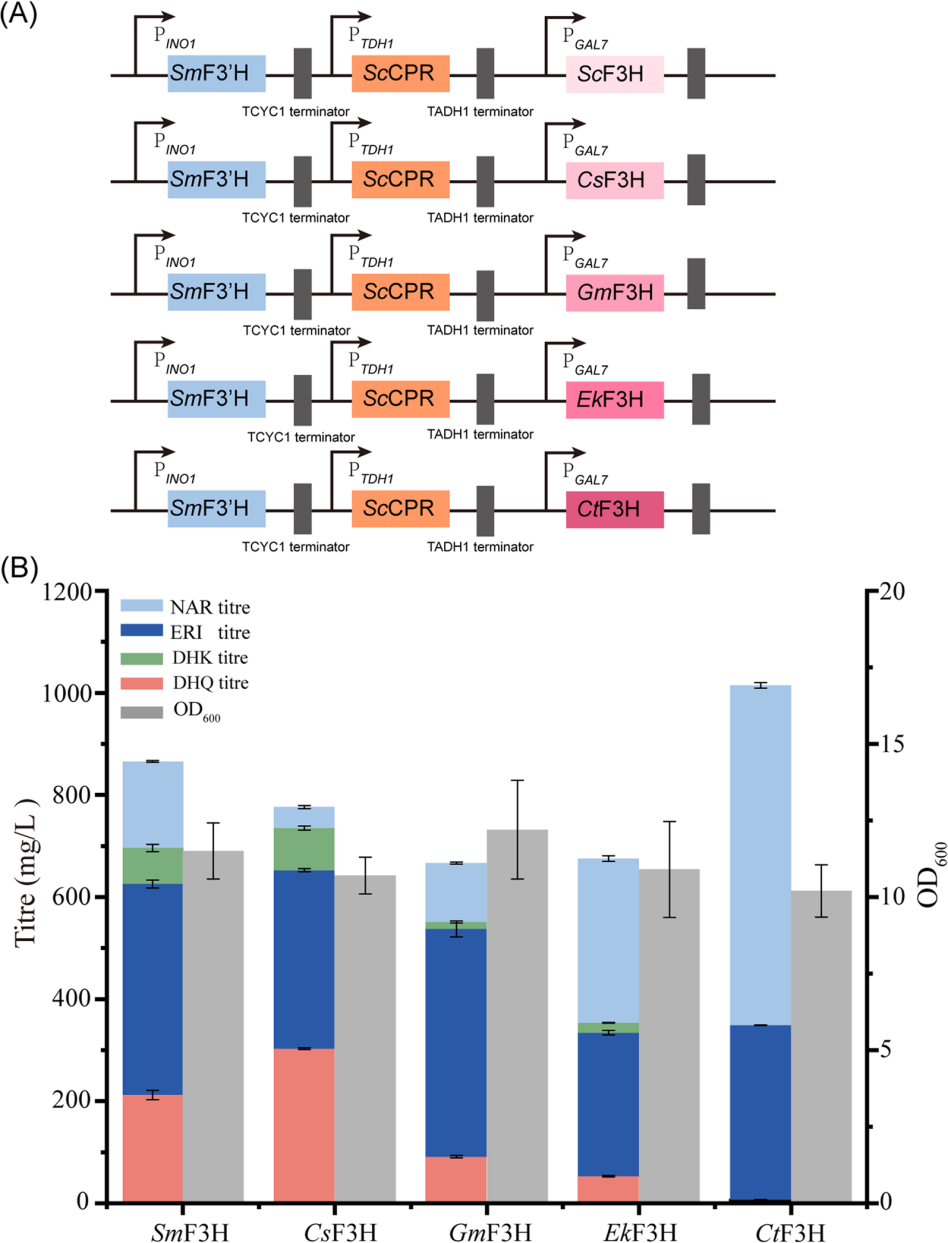
Selection of optimal F3H for dihydroquercetin production. (**A**) Plasmid construction for the co-expression the enzymes of F3Hs from different plant species with *Sm*F3′H and *Sc*CPR. The expression of F3Hs was regulated by the inducible promoter P_*GAL7*_. (**B**) Activities tests for F3Hs by determining the NAR biotransformation in flasks. Strains were cultivated in YPD supplemented with 1 g/L of NAR as the substrate. All experiments were performed in triplicates and error bars indicate SD

The enzyme F3H is capable of hydroxylating the compounds on the B-rings of NAR or DHK at the 3-positions. Five different F3Hs were cloned from different plant species enriched with flavonoids and relevant compounds, including *S. marianum* (*Sm*F3H), *Citrus sinensis* (*Cs*F3H), *Epimedium koreanum Nakai* (*Ek*F3H), *G. max* (*Gm*F3H), and *Carthamus tinctorius* (*Ct*F3H). To test their activities, these F3Hs were inserted into an expression vector containing F3′H and *Sc*CPR, and episomally expressed under the control of P_*GAL7*_ in *S. cerevisiae* (Fig. 3A). After 72-h fermentation, the recombinant strain expressing *Cs*F3H yielded 303.0 ± 1.7 mg/L of DHQ and a yield of 66.0 ± 3.7 mg/g_CDW_, the highest titre and yield in all tested groups (Fig. 3B). The recombinant strain expressing *Sm*F3H also showed relatively higher activies in the NAR conversion reaction, but the strain incorporating *Ct*F3H showed the worst performance in producing DHQ. A small amount of the substrate NAR was detected in the supernatant, but a significant amount of intermediate metabolites were detected, including 413.3 mg/L of ± 7.8 ERI and 70.4 ± 7.2 mg/L of DHK (Fig. 3B).

### Enhancement of dihydroquercetin production by promoter adjustment

**Fig. 4:**
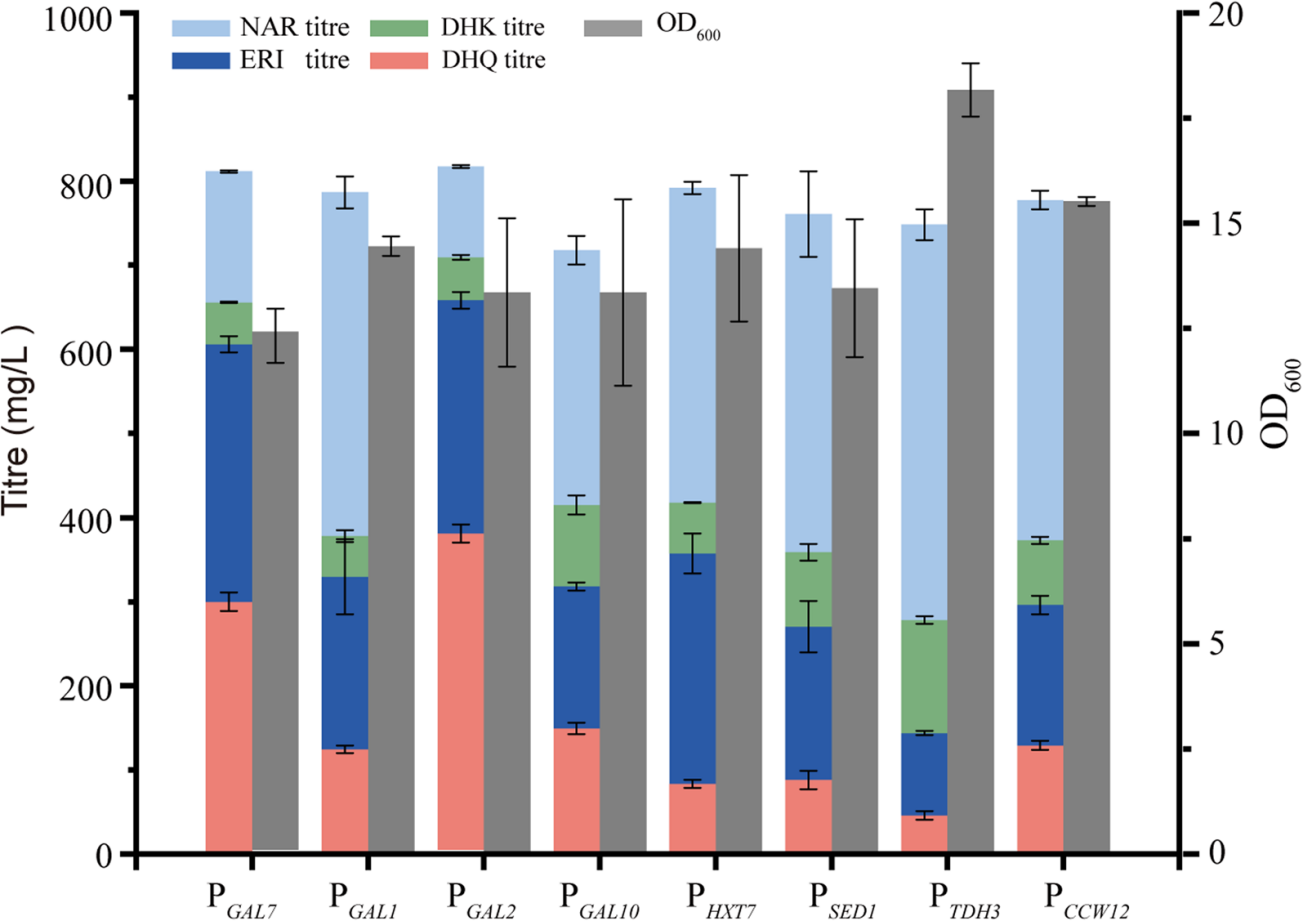
Enhancement of DHQ production by promoter adjustment. The original promoter of P_*GAL7*_ was replaced with different promoters, including the constitutive promoters of P_*SED1*_, P_*TDH3*_, P_*CCW12*,_ and P_*HXT7*_ and inducible promoters of P_*GAL1*_, P_*GAL2*_, and P_*GAL10*_. Strains were co-expressed with the *Sm*F3′H, *Sc*CPR and *Cs*F3Hs in the YPD medium supplemented with 1 g/L of NAR for DHQ production tests in the flasks. All experiments were performed in triplicates and error bars indicate SD

The above study showed that a considerable amount of ERI accumulated during the NAR biotransformation, indicating the inadequate conversion of ERI to DHK. Several factors may influence F3H expression and function, such as possible interference between F3H and F3′H due to competition for limited resources in cells, promoter strength and enzyme expression variation as a result of environmental stress and pH shift [33]. Considering these possibilities, the constitutive or inducible pomoters with different strengths, such as P_*SED1*_, P_*TDH3*_, P_*CCW12*_, P_*GAL1*_, P_*GAL2*_, P_*GAL10*_, and P_*HXT7*_, were used to control F3H expression in *S. cerevisiae*, and episomal expression cassettes for F3H were created utilizing these promoters (Fig. 4A). The recombinant strains were conducted following the fermentation protocol described previously, and the promoter P_*GAL2*_ worked best in all the tested groups. The recombinant strain using P_*GAL2*_ for regulating the F3H expression, accumulated 381.2 ± 10.7 mg/L of DHQ from 1 g/L of NAR with a yield of 67.0 ± 6.7 mg/g_CDW_ and a conversation of 0.34 ± 0.005 mol_DHQ_/mol_NAR_, improving by 27.1% compared with the parental strain using the Gal7 promoter (Fig. 4B). Surprisingly, the strong promoters P_*SED1*_, P_*TDH3*_, and P_*CCW12*_ performed poorly in this study, and a significant amount of substrate NAR was detected, indicating interference on F3′H catalysis. Another interesting finding is about the using the promoter P_*HXT7*_, a glucose-dependent promoter induced at a low level of glucose, resulted in significant ERI accumulation but low DHQ formation (Fig. 4B).

## Discussion

Dihydroquercetin is a bioactive flavonoid that has piqued the interest of industries and metabolic engineering communities due to its various health benefits. Dihydroquercetin bioproduction is an appealing alternative to fulfill the increased demand. Currently, DHQ has been de novo synthesized from glucose or glycerol. The titre and yield were only around 330 mg/L in *S. cerevisiae* in a bioreacto*r* [17] and about 110 mg/L in *Y. lipolytica* at shake flask level [15]. The titre and yield are low, partly due to considerable metabolite intermediator accumulation and low efficiency in the long pathway for de novo DHQ biosynthesis, which may need deep metabolic engineering to balance modules and efficiently direct the flux for DHQ synthesis as well as reduce metabolic burdens. The high-value-added DHQ can be manufactured using the low-cost substrate NAR, extracted from fruit peel waste. Several strategies were used in this study to improve the conversion of NAR to DHQ, including optimizing the interaction between F3'H and its redox partner, increasing F3'H enzyme stability, selecting optimal F3H, and temporal regulation to the performance of two important enzymes. In addition to being a viable option for de novo DHQ bioproduction, the engineered whole-cell biocatalyst can be applied to DHQ bioproduction by converting the relative cheap substrate NAR to value-added product.

The biosynthesis of DHQ involves hydroxylation of the flavonoid's B-ring at the 3′-position, which is catalyzed by the cytochrome P450-dependent flavonoid 3′-hydroxylase and needs the transfer of electrons to F3'H by the cytochrome P450 reductase. The P450-dependent F3'H and CPR form a 1:1 functional P450-reductase complex for the catalytic reaction, but the P450 enzyme and reductase partner may not be well balanced [34]. Furthermore, F3'H and *Sc*CPR have been identified as membrane-bound proteins, and optimizing expression levels will allow for better use of the limited membraneous space and other bioresources for protein synthesis. Previous studies have shown that adjusting the gene copies or promoter strengths to change the relative expression levels of F3'H and CPR was effective for improving DHQ production [15, 23]. When a P450 enzyme has a higher affinity for interacting with reductase, electrons will flow to those P450s first [35]. As a result, it is envisaged that strengthening the interaction between F3'H and its reductase partner would aid in accelerating the process. Our study demonstrated that the F3'H from *S. marianum* (*Sm*F3'H) and CPR from *S. cerevisiae* (*Sc*CPR) formed a functional reductase-P450 chimeric complex that performed best in the NAR conversion test. Further truncation studies revealed that the transmembrane region was critical for *Sm*F3'H function maintenance, partly because the catalytic reaction needs the membrane as the matrix for interaction and reaction, and enzyme organization and function are modulated by membrane components [36, 37].

The possibility of altering the potential ubiquitination sites is another way of enhancing DHQ synthesis. This strategy increases the key enzymes' half-lives, which may help to increase protein stability and, in turn, increase the production of the target compound. In a previous study, our team showed that altering the sensitive ubiquitination site of *Fj*TAL can significantly increase the synthesis of *p*-coumaric acid [32]. In this study, we conducted a test for the *Sm*F3'H and mutated the sensitive ubiquitination sites, leading to a slight increase in DHQ production. However, other mutations led to a reduction in enzyme activity because they might happen in the regions where catalytic activities or interactions with redox partners occur. In addition, cells monitor and get rid of misfolded or other aberrant proteins via the ubiquitin–proteasome pathway [38, 39], and the misfolded *Sm*F3'H may form aggregates and fail to be removed in time, which may cause some interference for cells. Fortunately, there is no obvious cell growth impairment that occurred in our study.

Efforts are also being undertaken to improve the activity and expression of F3H, the major enzyme responsible for hydroxylating the C-ring of flavonoids at the 3-position. Five F3Hs were selected from different plant species in our study, the F3Hs from *Petroselinum crispum* and *A. thaliana* were characterized to have significant affinity for NAR but were not chosen due to low activity in the mild pH range [40, 41]. Different types of promoters with different strengths were selected for evaluating their influence in DHQ production. Interestingly, we discovered that using strong promoters P_*SED1*_, P_*TDH3*_, and P_*CCW12*_ [42] resulted in a significant decrease in DHQ formation, whereas several promoters related to galactose catabolism or regulation worked better than those three. This implies that *Sm*F3'H/CPR expression and/or function is being interfered with. Different F3'Hs and F3Hs may exhibit a distinct variance in catalytic efficacy for diverse substrates and have different preferences for certain substrates [43]. It seems that the *Sm*F3'H prefers to use the substrate NAR rather than DHK, which means the DHQ is mainly via the route of ERI converted from NAR. In this scenario, the strong constitutive expression of F3H may compete for limited resources for *Sm*F3'H/CPR expression and overwhelm cell capacity in enzyme synthesis, while the use of weak constitutive promoter P_*HXT7*_ does facilitate NAR conversion to ERI but with low DHQ production. To avoid such possible interference, we employed the inducible promoters of P_*GAL1*_, P_*GAL2*_, P_*GAL10*,_ and P_*GAL7*_ to regulate F3H expression. By combining all of these strategies, the DHQ conversion was significantly improved, compared with the previous studies.

Different strategies for strain engineering and fermentation optimization were used in the literature, resulting in DHQ production in *S. cerevisiae* at around 330 mg/L in a bioreactor after 108 h of fed-batch fermentation, three folds higher than that in a flask [17], the currently reported highest titre. In another yeast, *Y. lipolytica*, the strain was engineered to produce 110 mg/L DHQ with high intermediator accumulation in the shake flask [15]. In our study, different strategies were applied to investigate the effectiveness, which produced 381.2 mg/L DHQ from NAR in deep-well microplate. Note that a much higher density can be achieved in a bioreactor than in a deep-well microplate, probably 9–10 folds. Furthermore, the optimization of fermentation conditions can further improve DHQ production. Therefore, the engineered strain for NAR biotransformation is likely more efficient in DHQ production in our study. However, more studies should be performed in the well-controlled bioreactor for comparison in the future.

## Conclusions

In conclusion, *S. cerevisiae* was engineered to produce dihydroquercetin from naringenin in this study. The key enzyme F3′H and its P450 reductase partner from different species were co-expressed in *S. cerevisiae*, and the *Sm*F3′H/*Sc*CPR formed the best P450-reductase enzyme complex to produce 435.7 ± 7.6 mg/L of ERI from NAR with a conversion rate of 0.41 ± 0.007 mol_ERI_/ mol_NAR_. The production formation was further improved by mutating the predicted potential ubiquitination sites for enhancing the *Sm*F3′H stability. Five different F3Hs from different plant species were co-expresed with *Sm*F3′H/*Sc*CPR for DHQ production, and could produced 381.2 ± 10.7 mg/L of DHQ from 1 g/L of NAR by further promoter replacement. In comparison to earlier research, the titre and yield of DHQ production was significantly improved by combining all of these strategies. This study provides systematic strategies for improving DHQ production from NAR.

## Supplementary Information

**Additional file 1: Table S1.** Genes were synthesized in this study. **Table S2.** Primers used in this study. **Fig. S1.** Influence of truncation the *Sm*F3′H and *Sc*CPR.

## Data Availability

All data and materials are available as described in the study and its Additional file 1.

## Abbreviations

DHQ: Dihydroquercetin
ERI: Eriodictyol
DHK: Dihydrokaempferol
NAR: Naringenin
F3′H: Flavonoid 3′-hydroxylase
F3H: Flavanone 3-hydroxylase
CHS: Chalcone synthase
CPR: Cytochrome P450 reductase
CRISPR: Clustered regularly interspaced short palindromic repeat
