# Supplementary material for: Engineering Saccharomyces cerevisiae for the production of dihydroquercetin from naringenin
Source: Microb Cell Fact. 2022 Oct 15;21:213. doi: 10.1186/s12934-022-01937-8 (PMC9569186; doi:10.1186/s12934-022-01937-8)
Supplement: Supplementary file 1 — Additional file 1: Table S1. Genes were synthesized in this study. Table S2. Primers used in this study. Fig. S1. Influence of truncation the SmF3′H and ScCPR. [file 12934_2022_1937_MOESM1_ESM.docx]

**Engineering *Saccharomyces cerevisiae* for the production of dihydroquercetin from naringenin**

Shiqin Yu^1,2,3,4^, Mingjia Li^1,2,3,4^, Song Gao, Jingwen Zhou^1,2,3,4,*^

^1^ Science Center for Future Foods, Jiangnan University, 1800 Lihu Road, Wuxi, Jiangsu 214122, China

^2^ Key Laboratory of Industrial Biotechnology, Ministry of Education and School of Biotechnology, Jiangnan University, 1800 Lihu Road, Wuxi, Jiangsu 214122, China;

^3^ Engineering Research Center of Ministry of Education on Food Synthetic Biotechnology, Jiangnan University, 1800 Lihu Road, Wuxi, Jiangsu 214122, China.

^4^ Jiangsu Province Engineering Research Center of Food Synthetic Biotechnology, Jiangnan University, 1800 Lihu Road, Wuxi, Jiangsu 214122, China.

^*^ Corresponding author：Jingwen Zhou

Mailing address: Science Center for Future Foods, Jiangnan University, 1800 Lihu Road, Wuxi, Jiangsu 214122, China.

Phone: +86-510-85914371, Fax: +86-510-85914371

E-mail: zhoujw1982@jiangnan.edu.cn

Table S1 Genes were synthesized in this study

|  | Sources | Reference |
| --- | --- | --- |
| **Genes** |  |  |
| *Sm*F3′H* | *Silybum marianum* | [1] |
| *Fa*F3′H | *Fragaria* *x ananassa* | AEE60885.1 |
| *Sm*CPR* | *Silybum marianum* | [1] |
| *At*CPR | *Arabidopsis thaliana* | X66017.1 |
| *Eb*CPR | *Erigeron breviscapus* | [2] |
| *Gm*CPR | *Glycine max* | XM_003541568.3 |
| *Ht*CPR | *Helianthus tuberosus* | Z26250.1 |
| *Sc*CPR | *Saccharomyces cerevisiae* | NP_011908.1 |
| *Sm*F3H | *Silybum marianum* | [3] |
| *Cs*F3H | *Citrus sinensis* | MH208416 |
| *Gm*F3H | *Glycine max* | NM_001249868.3 |
| *Ek*F3H | *Epimedium koreanum Nakai* | MT547762 |
| *Ct*F3H | *Carthamus tinctorius* | JF737995.1 |

Notes: * indicates that gene was cloned from reverse transcription

Table S2 Primers used in this study

|  | Sequence |
| --- | --- |
| D03-1-F | GCAATTAACCCTCACGCTTGTCAGACATCAGCGATC |
| D03-1-R | CTTAACTATGCGGCATCAGAGCAG |
| D03-2-F | GTCTGACAAGCGTGAGGGTTAATTGCGCGCTTG |
| D03-1-F | GCAATTAACCCTCACGCTTGTCAGACATCAGCGATC |
| D03-1-R | CTTAACTATGCGGCATCAGAGCAG |
| D03-2-F | GTCTGACAAGCGTGAGGGTTAATTGCGCGCTTG |
| D03-2-R | TCTCAGTACAATCTGCTCTGATGCC |
| D04-1-R | CGGAGATGGAGTCTCTTCATCTTCTTCGTCACG |
| D04-2-F | GAAGATGAAGAGACTCCATCTCCGATTCGATTC |
| D05-1-R | GATTTCGATATCTGAAAGTCTCCCGCCCTCTCCATTGG |
| D05-2-F | GGCGGGAGACTTTCAGATATCGAAATCAAAGCTTTGC |
| D06-1-R | GAATCGGAGATGGAGTTTTCTCATCTTCTTCGTCACGCTTTG |
| D06-2-F | GAAGATGAGAAAACTCCATCTCCGATTCGATTC |
| D07-1-R | GCGTCATGAACTCTCAAAAACTGTGCCG |
| D07-2-F | CACAGTTTTTGAGAGTTCATGACGCCAATTTCGC |
| D08-1-R | GCGTCATCTCTGAGCGAAATCAACGTGC |
| D08-2-F | CGTTGATTTCGCTCAGAGATGACGCCAATGGAG |
| D09-1-R | GATAATTATACGCGATGTGTCTCGCGCCGGAGTTCG |
| D09-2-F | GGCGCGAGACACATCGCGTATAATTATCAGG |
| D10-1-R | GGAGTTTCTTCATTCTCTTCGTCACGCTTTGC |
| D10-2-F | GTGACGAAGAGAATGAAGAAACTCCATCTCCG |
| D45-1-F | GATGGAGCCATCTTAATAGAGCGAACGTATTTTATTTTGC |
| D45-1-R | GCTGGCGTAATAGCCGACAACATTCCACCCAACAAC |
| D45-2-F | GAATGTTGTCGGCTATTACGCCAGCTGAATTG |
| D45-2-R | GTTGAGTGTTGTTCCAGTTTGG |
| D45-3-F | GTGGACTCTTGTTCCAAACTGG |
| D45-3-R | GTTCGCTCTATTAAGATGGCTCCATCTACTTTGACTG |
| D46-1-F | GATGGAGCCATTTTGTTTGTTTATGTGTGTTTATTCGAAAC |
| D46-1-R | CGTAATAGCCACGCTTTTTCAGTTCGAGTTTATC |
| D46-2-F | GAACTGAAAAAGCGTGGCTATTACGCCAGCTGAATTG |
| D46-3-R | CACATAAACAAACAAAATGGCTCCATCTACTTTGACTG |
| D47-1-F | GGAGCCATTATTGATATAGTGTTTAAGCGAATGACAG |
| D47-1-R | CAGCTGGCGTAATAGCTTCGCGGCCACCTAC |
| D47-2-F | GGCCGCGAAGCTATTACGCCAGCTGAATTG |
| D47-3-R | CTTAAACACTATATCAATAATGGCTCCATCTACTTTGACTG |
| D48N-1-F | GTAGATGGAGCCATAGCTCTGCTTATATAGACCTCCC |
| D48N-1-R | CTGGCGTAATAGCGACATTGATTATTGACTAGTTATTAATAGTAATCAATTAC |
| D48N-2-F | CTAGTCAATAATCAATGTCGCTATTACGCCAGCTGAATTG |
| D48N-2-R | CTATATAAGCAGAGCTATGGCTCCATCTACTTTGACTG |
| D49-1-F | GTAGATGGAGCCATTATAGTTTTTTCTCCTTGACGTTAAAGTATAG |
| D49-1-R | CAGCTGGCGTAATAGCGATTAGAAGCCGCCGAGC |
| D49-2-F | GCTTCTAATCGCTATTACGCCAGCTGAATTG |
| D49-2-R | GTTGAGTGTTGTTCCAGTTTGG |
| D49-3-F | GTGGACTCTTGTTCCAAACTGG |
| D49-3-R | GAGAAAAAACTATAATGGCTCCATCTACTTTGACTG |
| D50-1-F | GAGCCATTATGAAAGAATTATTTTTTTTATTATGTTAATCTTGTGTTTAC |
| D50-1-R | GCTGGCGTAATAGCCTCCTACAATACCAGTTTCGCTG |
| D50-2-F | GGTATTGTAGGAGGCTATTACGCCAGCTGAATTG |
| D50-3-R | CATAATAAAAAAAATAATTCTTTCATAATGGCTCCATCTACTTTGACTG |
| D51-1-F | GGAGCCATTTATATTGAATTTTCAAAAATTCTTACTTTTTTTTTGG |
| D51-1-R | GCTGGCGTAATAGCCCAGGTTACTGCCAATTTTTCC |
| D51-2-F | CAGTAACCTGGGCTATTACGCCAGCTGAATTG |
| D51-3-R | GAATTTTTGAAAATTCAATATAAATGGCTCCATCTACTTTGACTG |
| D52-1-F | GGAGCCATTTTTTGATTAAAATTAAAAAAACTTTTTGTTTTTGTG |
| D52-1-R | CAGCTGGCGTAATAGCGAAATGAGGGGTATGCAGGAATTTG |


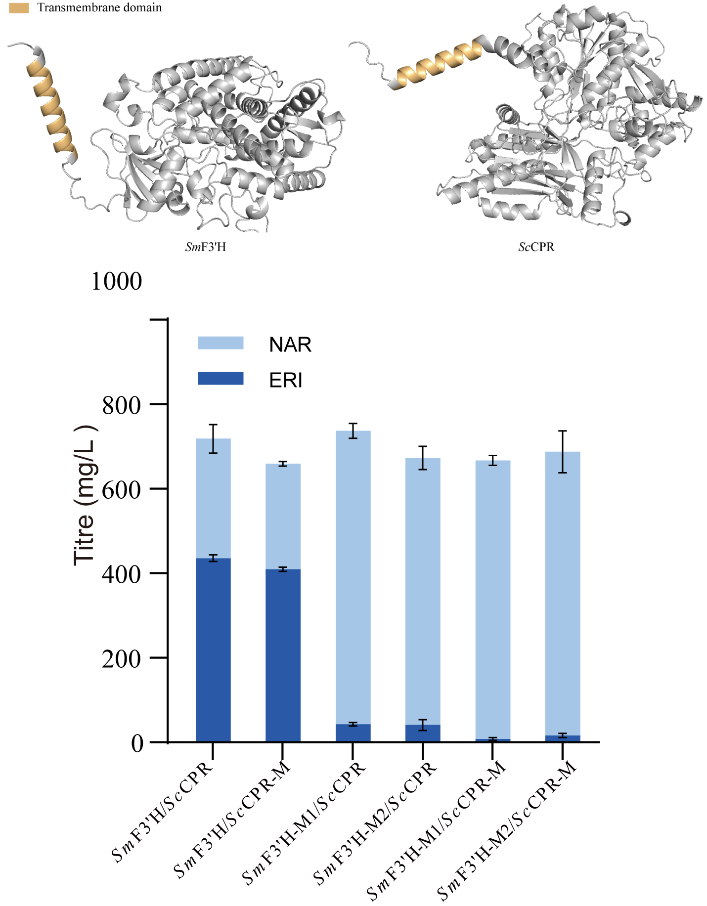


Fig. S1 influence of truncation the *Sm*F3′H and *Sc*CPR

1. Lv Y, Gao S, Xu S, Du G, Zhou J, Chen J: **Spatial organization of silybin biosynthesis in milk thistle [Silybum marianum (L.) Gaertn].** *Plant J* 2017, **92:**995-1004.

2. Liu X, Cheng J, Zhang G, Ding W, Duan L, Yang J, Kui L, Cheng X, Ruan J, Fan W, et al: **Engineering yeast for the production of breviscapine by genomic analysis and synthetic biology approaches.** *Nat Commun* 2018, **9:**448.

3. Gao S, Lyu Y, Zeng W, Du G, Zhou J, Chen J: **Efficient Biosynthesis of (2S)-Naringenin from p-Coumaric Acid in Saccharomyces cerevisiae.** *J Agric Food Chem* 2020, **68:**1015-1021.
